# Single-stage repair of contaminated hernias using a novel antibiotic-impregnated biologic porcine submucosa tissue matrix

**DOI:** 10.1186/s12893-020-00715-w

**Published:** 2020-03-30

**Authors:** Samuel Minor, Carl J. Brown, Paul S. Rooney, Jason P. Hodde, Lisa Julien, Tracy M. Scott, Ahmer A. Karimuddin, Manoj J. Raval, P. Terry Phang

**Affiliations:** 1 QE2 Hospital, QEII Health Sciences Centre Dalhousie University, 1278 Tower Road, Halifax, NS B3H 2Y9 Canada; 2 St. Paul’s Hospital, 1081 Burrard Street, Vancouver, BC V6Z 1Y6 Canada; 3 Royal Liverpool Hospital, Prescot Street, Merseyside, Liverpool L7 8XP UK; 4 Cook Biotech Incorporated, 1425 Innovation Place, West Lafayette, IN 47906 USA

**Keywords:** Hernia repair, Graft, Abdominal wall reconstruction, Contamination

## Abstract

**Background:**

Single-stage repair of incisional hernias in contaminated fields has a high rate of surgical site infection (30–42%) when biologic grafts are used for repair. In an attempt to decrease this risk, a novel graft incorporating gentamicin into a biologic extracellular matrix derived from porcine small intestine submucosa was developed.

**Methods:**

This prospective, multicenter, single-arm observational study was designed to determine the incidence of surgical site infection following implantation of the device into surgical fields characterized as CDC Class II, III, or IV.

**Results:**

Twenty-four patients were enrolled, with 42% contaminated and 25% dirty surgical fields. After 12 months, 5 patients experienced 6 surgical site infections (21%) with infection involving the graft in 2 patients (8%). No grafts were explanted.

**Conclusions:**

The incorporation of gentamicin into a porcine-derived biologic graft can be achieved with no noted gentamicin toxicity and a low rate of device infection for patients undergoing single-stage repair of ventral hernia in contaminated settings.

**Trial registration:**

The study was registered March 27, 2015 at www.clinicaltrials.gov as NCT02401334.

## Background

Surgical site infection (SSI) following synthetic mesh repair in contaminated fields is high, with rates of 30–42% being reported [1–3]. The use of synthetic materials for ventral hernia repair in contaminated fields has been controversial because a mesh infection may necessitate subsequent removal of the mesh in order to clear the infection [4–8]. Expert consensus and several systematic reviews recommend the use of biologic grafts when the risk of surgical site events is high, such as in the presence of gross contamination, because infection of biologic grafts can usually be managed conservatively without the requirement of surgical explantation [5, 9, 10]. However, infection has been postulated to be one of the main mechanisms for hernia recurrence [10, 11], with recurrence rates of around 30% being reported for ventral hernia repair in contaminated settings [1, 8, 12–19].

Porcine small intestinal submucosa (SIS) is a type of biologic hernia graft whose performance in contaminated settings have been previously described [20]. We postulated that by incorporating gentamicin sulfate into a SIS graft that we could protect it from bacterial colonization and prevent premature implant degradation secondary to bacterial protease activity. A small, multicenter, pilot study was designed to determine the incidence of surgical site infection following implantation of a gentamicin sulfate-containing SIS hernia graft during the repair of incisional hernias in surgical fields characterized as Centers for Disease Control and Prevention (CDC) Classes II, III, or IV. Secondary outcomes included the rate of hernia recurrence and other clinical and patient-reported outcomes.

## Methods

### Antimicrobial hernia repair device development

**Fig. 1:**
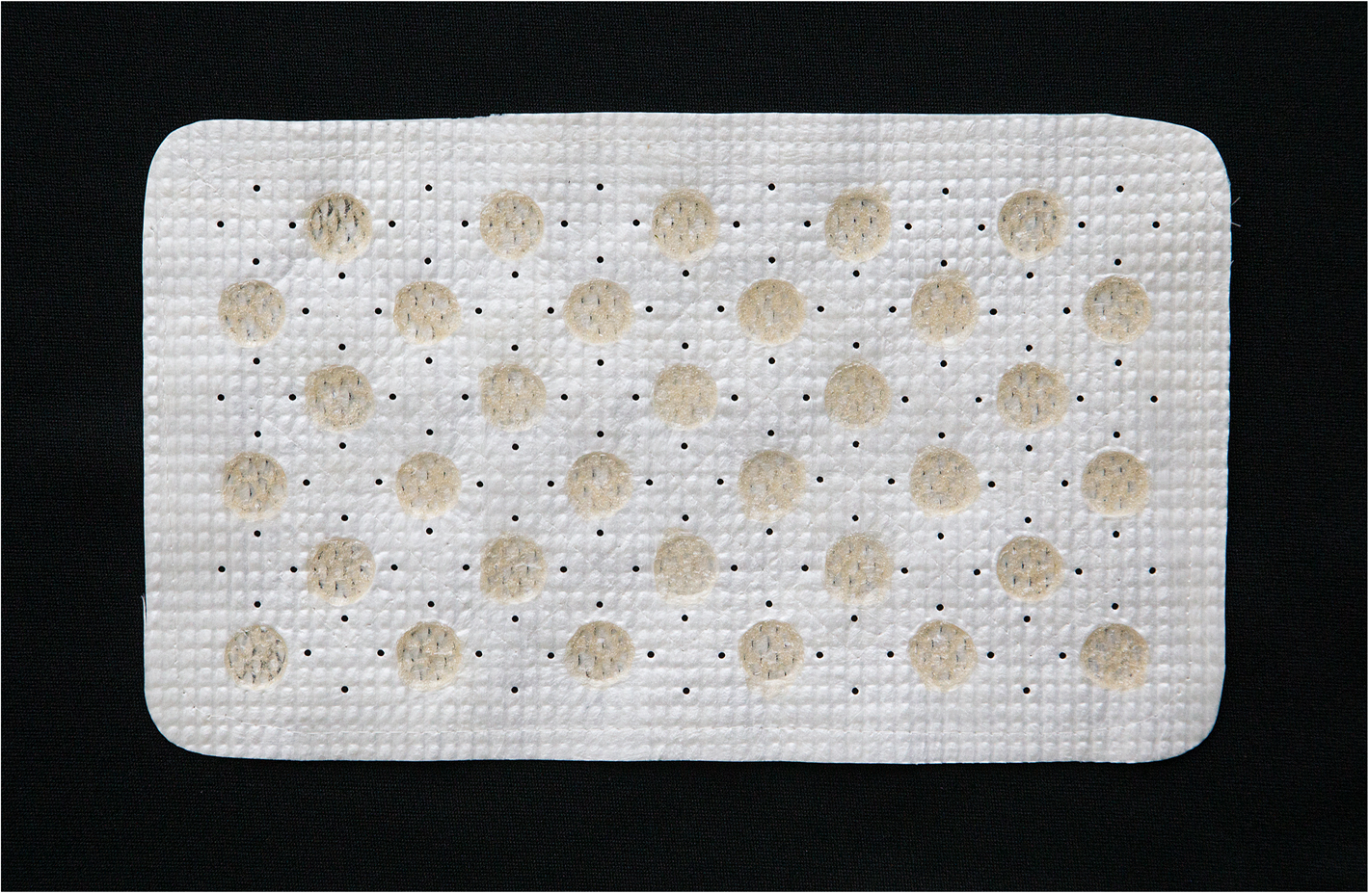
Antimicrobial Hernia Repair Device

The Antimicrobial Hernia Repair Device (AHRD) (Cook Biotech Incorporated, West Lafayette, IN, USA) consists of a textured, multilayered SIS lyophilized sheet with raised-walled craters of approximately 12 mm in diameter. A controlled volume of gentamicin sulfate suspension in a solution of poly-polyDL-Lactide-Co-Glycolide (PLGA) in acetone is deposited into the craters on the device and dried to form controlled-release disks each containing 4.56 mg antibiotic (Fig. 1). The disks are placed on one side of the graft only, the side oriented away from the bowel, to reduce the risk of the PLGA exacerbating adhesion formation to the viscera. During device development, plasma gentamicin was designed to peak at 4–6 h after implantation and then gradually decline over a span of 5 days. The device delivers the antibiotic locally at a peak minimal inhibitory concentration (MIC) > 10:1 with the average MIC exceeded for over 24 h and is therefore capable of delivering relatively high local doses of drug without causing systemic toxicity.

### Study design

This was a prospective, multicenter, single-arm pilot study designed to evaluate the safety and efficacy of the AHRD. The study was approved by the Institutional Review Boards of all participating institutions (Nova Scotia Health Authority Research Ethics Board, Providence Health Care Research Institute, and National Research Ethics Service Liverpool East) and adhered to the STROBE guidelines. All patients provided written informed consent and were recruited for study inclusion between August 2015 and February 2017. The study was registered at www.clinicaltrials.gov as NCT02401334.

A predefined number of up to 30 patients at 3 clinical sites in the United Kingdom and Canada was chosen as the sample size for this pilot study. The clinical sites were chosen from centres with previous extensive experience of using SIS in ventral hernia repair. No power calculation was performed, as there was no control arm and this study was a pilot study designed to gather initial outcomes data to inform a subsequent pivotal study. Patients were screened sequentially for eligibility at each site. Inclusion criteria included patients over 21 years old with a ventral or incisional hernia that was to be surgically corrected with open surgery. Patients were required to have a Class II, Class III, or Class IV surgical field at the time of the operation, as defined clinically using the investigator’s best judgement.

Patients were excluded from the study if they were pregnant, had a known allergy to porcine products, had a body mass index (BMI) < 25 kg/m^2^, body weight < 45 kg, glycosylated hemoglobin (Hgb A1c) > 10%, albumin < 2.5 g/dL or pre-albumin < 5.0 mg/dL. Patients were also excluded if they had an existing serum gentamicin level of > 2 mg/L on the morning of surgery or any of the following contraindications to gentamycin: renal insufficiency (as determined by an estimated glomerular filtration rate (GFR) < 60 mL/min/1.73 m^2^), allergy to aminoglycosides, or myasthenia gravis. Patients with dirty-infected (Class IV) surgical fields required pre-operative culture and sensitivity of the contaminating organism demonstrating sensitivity to gentamicin sulfate.

### Operative details

Peri- and post-operative patient care, including the use of peri-operative antibiotics, was conducted in accordance with surgeon discretion and institutional standard of care. However, if applicable, complete removal of all infected mesh was required during the same procedure prior to implanting the AHRD. Device placement required an overlap of greater than 4 cm in all dimensions, but the surgical plane into which the device was implanted was variable based on surgeon preference and individual patient requirements. Graft fixation was completed using interrupted unbraided sutures. Abdominal wall reconstruction required complete fascial coverage of the graft, using anterior components separation if necessary. Closed suction drains were utilized at the surgeon’s discretion. Primary skin closure was achieved in all patients. Perioperative antibiotics, method of skin closure and wound dressings, and the use of negative pressure therapy were at the discretion of the surgeon.

### Outcomes

The primary outcome of the study was the incidence of surgical site infection as defined by the Centers for Disease Control and Prevention. Secondary outcomes included other procedural and post-operative adverse events related to the device or to the procedure, and hernia recurrence through 1 year.

To determine the systemic absorption and effect of gentamicin on the patient, serum gentamicin levels were obtained 24 h following graft implantation and incidence of nephrotoxicity (as determined by immediate impairment of kidney function) was assessed. Other adverse events related to the device or to the procedure were also recorded. Patients were followed daily in hospital then and at 2 weeks, 1 month, 3 months, 6 months and 12 months. A Data Safety Monitoring Board (DSMB) consisting of independent physicians and statisticians who were not investigators in the study, nor had a real or perceived conflict of interest with the conduct and administration of the study, were convened on a regular basis to evaluate the clinical study progress and review adverse events.

### Statistical analysis

Continuous variables were summarized using means and standard deviations, and categorical variables were summarized with frequencies and percentages. All enrolled patients were used to describe the outcomes.

## Results

### Patient characteristics

Twenty-four patients signed the informed consent document at 3 different sites, were treated, and were included in the analysis. One patient withdrew consent after the first follow-up visit, but all other patients were followed for at least 12 months after the index procedure. General patient demographics are listed in Table 1. Patients with contaminated and dirty surgical fields comprised most of the cohort (Table 2), with one patient enrolled whose wound was reclassified to Class I after the procedure. This patient was not removed from the analysis because the initial clinical determination was Class II.

**Table 1:** Baseline patient demographics and risk factors/comorbidities

Age (yrs) (Mean ± SD)		56.4 ± 10.9
Average BMI (Mean ± SD)		34.0 ± 6.2
Sex	Female	58% (14/24)
	Male	42% (10/24)
Current smoker		25% (6/24)
Chronic obstructive pulmonary disease (COPD)		13% (3/24)
Immune system disease		4% (1/24)
Hypertension		33% (8/24)
Diabetes	Type I	4% (1/24)
	Type II	29% (7/24)
Intra-abdominal malignancy		17% (4/24)
Previous abdominal surgery		100% (24/24)

**Table 2:** Operative Information

**Surgical wound classification**	**% (n/N)**
Clean	4% (1/24)
Clean-contaminated	29% (7/24)
Contaminated	43% (10/24)
Dirty-infected	25% (6/24)
**Device size**	**% (n/N)**
20 × 30 cm	67% (16/24)
13 × 22 cm	33% (8/24)
**Device overlap**	**(cm ± SD)**
Left lateral	7.7 **±** 2.8
Right lateral	7.5 **±** 2.4
Caudal/inferior	5.4 **±** 1.9
Cephalad/superior overlap	5.0 **±** 1.8
**Device placement**	**% (n/N)**
Onlay	8% (2/24)
Intraperitoneal	67% (16/24)
Retrorectus/preperitoneal	25% (6/24)
**Other operative details**	**% (n/N)**
Removal of previous mesh	33% (8/24)
Components separation performed	79% (19/24)
Primary fascial closure	100% (24/24)
Closed suction drains placed	92% (22/24)
**Operation duration, min (mean (range))**	265 (85–565)

Most defects were incisional hernias (96%, 23/24) and 58% (14/24) were classified as recurrent in nature. The average hernia length was 17.1 ± 5.9 cm and the average fascial separation was 13.2 ± 5.1 cm wide. Dimensions were determined radiographically or by direct measurement intra-operatively. Primary fascial closure over the graft was achieved in all patients with an overlap of greater than 4 cm on the lateral and caudal/inferior margins; two patients were implanted with only a 3 cm overlap at the cranial/superior edge because the length of the hernia extended right from the xiphiod process to the pubic bone. To achieve fascial apposition, anterior components separation using lateral relaxing incisions was performed in 19 patients (79%) and the remaining patients had primary closure of the mesh without a component separation. Repairs were performed using grafts measuring 20 × 30 cm (67%) and 13 × 22 cm (33%). The device was trimmed to fit in 63% of patients. The device was placed intraperitoneal in 63%, retrorectus in 29% and onlay in 8% of patients. Closed suction drains were used in the subcutaneous space in 20 patients (83%) and directly on the graft in 9 (38%). The average serum gentamicin at 24 h was 0.62 ± 0.4 mg/L (0.2–1.31 mg/L). The recommended therapeutic serum trough level of gentamicin is less than 2 mg/L.

### Wound events

At 12 months post repair, 5 patients had experienced 6 surgical site infections (21%). Deep surgical site infection involving the graft occurred in 2 patients (8%). Superficial infections, not involving the graft, were experienced by 3 patients (13%). One patient had a deep surgical site infection reported at both 2 weeks and 3 months post-operatively. The majority of patients with surgical site infections occurred in the first 2 weeks (3/5 (60%)) and only one infection occurred at 3 months post-operatively. Seromas were reported in 8 patients (33%). Wound dehiscence occurred in one patient (4%). Deep infections were managed with percutaneous drainage and IV antibiotics. Overall, 10 patients (42%) experienced at least one surgical site occurrence. No graft explantation or debridement was required.

### Other adverse events

In addition to the wound events presented above, a total of 35 additional adverse events related to the hernia repair procedure were reported, affecting 23 patients. The summary of these adverse events is presented in Table 3. Hernia recurrence occurred in 6 (25%) patients through 12 months. One recurrence was noted at 3 months, 2 more at 6 months and another 2 at 12 months. Of the 6 recurrences reported, 2 patients also had an infectious complication, 1 of which was a deep space surgical site infection. One of the recurrences occurred in a patient who presented with a post-operative seroma, intraabdominal abscess, and fistula formation. One incident of nephrotoxicity was reported, which was thought to be related to hypovolemia. This patient presented with a baseline GFR at the time of the procedure of 62 mL/min/1.73 m^2^ and a GFR 24 h post-procedure of 24 mL/min/1.73 m^2^. The patient was supported medically, and kidney function recovered. There were no deaths reported.

**Table 3:** Other hernia-related adverse events

Adverse Event	Total, % (n/N)
Abscess	17% (4/24)
Abdominal pain (transient)	21% (5/24)
Bowel obstruction	4% (1/24)
Fistula	8% (2/24)
Hematoma	4% (1/24)
Hernia recurrence	25% (6/24)
Ileus	17% (4/24)
Incisional pain	4% (1/24)
Nephrotoxicity	4% (1/24)
Seroma	33% (8/24)
Wound dehiscence	8% (2/24)

## Discussion

This is the first clinical study to describe the incorporation of local antibiotics into an SIS biologic hernia graft. The study demonstrates that the addition of gentamicin into a biologic device is safe, with low measured systemic gentamicin levels at 24 h, and has a rate of graft infection that is not consistent with reported rates in the literature. This study may encourage further study into graft construction utilizing high levels of local antibiotics in contaminated settings.

Deep surgical site infection directly involving the AHRD is not consistent with what has been previously described for non-antibiotic impregnated biologic grafts when placed in patients at high risk of infection. However, this finding closely mirrors the findings of a single arm study using a rifampin/minocycline-coated, non-crosslinked porcine acellular dermis, in which implant of the device in complex abdominal wall reconstruction patients was associated with a low 30-day rate of postoperative surgical site occurrences/postoperative complications [21].

Table 4 summarizes studies utilizing biologic grafts in single-stage repair in contaminated fields and describes infection rates 2 to 3 times higher than what was observed in the current study. A recent systematic analysis reported that the pooled infection rate for biologic graft materials placed in contaminated fields was 35.5% [1].. Using data from Kanter’s series of ventral hernia repair [27], an infection rate of approximately 55% would be predicted in this study’s patient mix of clean-contaminated, contaminated and dirty patients. Three previous case series have described the use of SIS in contaminated fields. Madani et al. [19] placed SIS in 46 patients with clean-contaminated (35%), contaminated (24%) and dirty (41%) fields and reported a 30% rate of graft infection. They also reported that all infected grafts required re-operation and explantation. Ueno et al. [26] utilized SIS in 18 ventral hernia repairs with potentially contaminated (50%) and grossly contaminated fields (50%). In their series, they reported a 40% infection rate and one patient in which the graft was completely degraded by the infection. Helton et al. [11] described SIS hernia graft use in 31 patients with dirty and clean-contaminated fields, with 13 (42%) patients experiencing infections involving the graft [28]. It is clear that biologic grafts are not more resistant to infection over other materials. However, direct comparison of this antibiotic impregnated graft with these retrospective studies is not possible and this study only serves to generate future hypothesis.

**Table 4:** Review of studies utilizing biologic grafts in repair of ventral hernias with clean-contaminated, contaminated, and dirty fields

Paper	Year	n	Infection %
Atema JJ, et al. [15]	2016	Review	20–75
Atema JJ, et al. [22]	2017	80	45
Helton WS, et al. [11]	2005	53	42
Itani KMF, et al. [18]	2012	80	30
Chamieh, et al. [17]	2017	34	50
Kaufmann R, et al. [23]	2019	77	27
Lee L, et al. [1]	2014	Review	32 (clean contaminated)
			36 (contaminated and dirty)
Madani A, et al. [19]	2017	46	54
Majumder A, et al. [16]	2016	69	32
Rosen MJ, et al. [14]	2013	128	48
Shubinets V, et al. [8]	2018	Review	30
Slater NJ, et al. [24]	2013	Review	19
Wind J, et al. [25]	2009	Review	35
Ueno T, et al. [26]	2004	20	40

Many of the studies outlined in Table 4 describe the requirement for graft explanation or partial debridement [11, 18, 26]. No graft debridement or surgical explantation was required in our study.

The rate of hernia recurrence was 25% in this study and was similar to other reports that used SIS in contaminated fields with reported recurrence rates of 26% at 1 year [11], 30% at 15.7 months [26] and 43% at 47 months [19]. We hypothesized that a lower rate of graft infection would be associated with a lower hernia recurrence rate, but this was not demonstrated. Bacterial degradation of the biologic graft in the setting of infection has been postulated to be a major factor contributing factor to hernia recurrence. However, our study suggests that there may be other, more dominant factors than infection that affect hernia recurrence. Other studies have also failed to demonstrate an association between infection and hernia recurrence [26]. Although the reduction of graft infection is desirable in terms of requirements for repeat surgical procedures, wound care and systemic antibiotics, it does not appear to be the panacea for hernia recurrence. Further research is required to explore the mechanisms behind hernia recurrence in order to direct innovations to mitigate them.

The observed gentamicin level at 24 h in this study was well below levels associated with toxicity (trough level < 2 mg/L), and no patient experienced a complication directly attributable to gentamicin. One patient had renal impairment that was felt to be secondary to hypovolemia. There was a high rate of overall complications, with almost all patients having at least one complication. This is similar to other studies of single-staged repair in contaminated settings and speaks to the complex and challenging nature of these cases.

This study is one of the few prospective, multi-center studies examining the repair of incisional hernias in contaminated settings. The majority of reports on this patient population are retrospective and are limited to a single institution or single-surgeon experience. To our knowledge, the only other prospective study in this patient population, the RICH study, examined the performance of a non–cross-linked, porcine, acellular dermal matrix [18].

There are multiple limitations of this study including lack of comparative group, lack of blinding, randomization and relatively short follow-up of 12 months. Long term follow-up of repairs using porcine acellular dermis demonstrate recurrence rates increasing out to 3 years, with a median time to recurrence of over 2 years [14]. Our sample size of 24 is small, although is of average size for studies examining this patient population. There is the potential for selection bias, as this study does not describe what patients declined to be involved in the study or how the surgeons selected patients for surgery. Additionally, 25% of the patients were current smokers, which is known to have a negative effect on outcomes, and one patient of CDC Class I was included against the inclusion criteria. This study was also industry sponsored with its inherent potential for bias, however there are no plans to move forward with this prototype device to market.

We acknowledge that comparative clinical studies need to be completed before any recommendations can be made regarding what type of graft should be utilized in contaminated settings. Furthermore, the use of light-weight permanent mesh in the retrorectus space is being described more frequently in the literature as an option for single-staged repairs [1, 17]. Future studies should examine the incorporation of local antibiotics into this setting.

## Conclusion

In conclusion, the incorporation of gentamicin into an experimental porcine derived submucosa biologic graft can be performed with no noted toxicity from the gentamicin and with an 8% incidence of graft infection for patients undergoing single-stage repair of ventral hernia in contaminated settings. This study of a novel device may encourage future study into incorporating local antibiotics into biologic hernia grafts.

## Data Availability

The datasets used and/or analysed during the current study are available from the corresponding author on reasonable request.

## Abbreviations

SIS: porcine small intestine submucosa
AHRD: Antimicrobial Hernia Repair Device
